# Epidemiological patterns and occupational predictors of electrocardiographic abnormalities in seafarers: a multicenter health screening analysis

**DOI:** 10.3389/fpubh.2025.1602761

**Published:** 2025-10-15

**Authors:** Can Cui, Hui Zhu

**Affiliations:** Department of Health Medicine, General Hospital of The Yangtze River Shipping, Wuhan Brain Hospital, Wuhan, China

**Keywords:** seafarers, ECG abnormalities, risk factors, health management, marine safety

## Abstract

**Background:**

To analyze the results of seafarers’ health checkups, to understand the physical health status of seafarers and the high incidence of diseases, and to investigate the factors related to the abnormalities of electrocardiograms (ECG) of seafarers, so as to provide theoretical basis for improving the health level of seafarers and formulating the health intervention plan for seafarers.

**Methods:**

10,174 seafarers who underwent health checkups at the Yangtze River Shipping General Hospital from January 2024 to December 2024 were selected as the survey subjects, and clinical data such as their general conditions, ECG and other functional examinations, and biochemical indicators were extracted. SPSS 26.0 software was used for statistical analysis to understand the disease composition of seafarers’ health checkups, and single-factor analysis and multifactor logistic regression analysis were used to explore the influencing factors of seafarers’ abnormal ECG results.

**Results:**

Abnormal body mass index (BMI), abdominal obesity, refractive error, liver function abnormality, ECG abnormality, high blood pressure, high fasting blood glucose and other abnormalities were more common in the health checkup of seafarers. Among them, abnormal ECG is mainly classified into myocardial ischemia/infarction and arrhythmia. Fatty liver and urinary stones were common in ultrasound examination of seafarers over 40 years old. High blood pressure, high fasting blood glucose and smoking were risk factors for abnormal ECG results.

**Conclusion:**

Carrying out the health management of seafarers, strengthening the health education of seafarers with high prevalence of diseases, dynamically tracking the abnormal results of seafarers’ physical examination, and increasing the prevention and control of cardiovascular diseases in seafarers’ group can effectively improve the comprehensive health level of seafarers’ group.

## 1 Background

Maritime transport constitutes a critical component of global economic infrastructure and international logistics networks. With the maritime workforce expanding rapidly, occupational health management for seafarers has become an urgent public health priority. This is underscored by documented cases of preventable mortality due to delayed treatment of acute conditions during voyages, which impacts both individual health outcomes and industry sustainability. We conducted a retrospective analysis of 10,174 seafarers undergoing routine medical examinations at Yangtze River Shipping General Hospital (Wuhan, China) between January and December 2024. This cross-sectional study characterizes prevalent disease patterns and their demographic determinants, aiming to establish targeted health monitoring frameworks and improve maritime occupational health services.

## 2 Methods

### 2.1 Study design and population

This study focused on the Chinese professional seafarer population, encompassing both Yangtze River inland seafarers and ocean-going seafarers. All participants were required to undergo mandatory occupational health examinations at medical institutions designated by the Maritime Safety Administration of China after completing the statutory disembarkation procedures. No restrictions were imposed on the participants’ places of origin; their transportation methods (such as public transport, private vehicles, etc.) and related expenses were borne by individuals themselves. The examinations were initiated after seafarers had completed their voyage tasks and formally disembarked. These examinations might be conducted on the day of disembarkation or within the subsequent rest days, rather than being compulsorily limited to the first 24 h after disembarkation, ensuring that the seafarers’ physical conditions were in a relatively basal state. All Chinese seafarers underwent identical nationally standardized examinations (GB 30035–2013). Foreign seafarers follow flag state-nationality agreements; none were examined at our institution. However, most of included Chinese seafarers served international routes (Trans-Pacific, Asia-Europe, Southeast Asia trunk lines), whose occupational exposure profiles encompass global shipping risks, ensuring cross-cultural generalizability. Epidemiological data indicate that female seafarers globally constitute only 1% of the maritime workforce, with a further reduced proportion in deep-sea positions (predominantly concentrated in service-support roles). Based on the principles of population representativeness and requirements for homogeneity of exposure, this study has enrolled 10,174 Chinese male seafarers who underwent physical examinations at Yangtze River Shipping General Hospital between January and December 2024 as research participants. The core indicators required for this study (such as gender, age, BMI, etc.) are all mandatory items. Their physical examination data are accompanied by corresponding serial numbers and original records. During research data entry, logical consistency checks were conducted on relevant indicators using SPSS 26.0 to eliminate entry errors. The inclusion criteria for study participants were as follows: (i) Age: 18–60 years (encompassing the active seafaring workforce); (ii) Complete dataset: Anthropometric (height, weight), clinical (blood pressure), and laboratory measures; (iii) Chinese nationality with registration in mainland China’s maritime authorities (excluding Hong Kong, Macao, and Taiwan); (iv) Primary occupation: Maritime transport employment (≥50% annual income). Participants meeting all criteria were enrolled; others were excluded.

### 2.2 Research methods

This study employed a retrospective design, extracting data from the health examination system of Yangtze River Shipping General Hospital’s Physical Examination Center. To ensure data quality, researchers implemented standardized training protocols for all personnel handling data extraction, verification, and entry. The dataset comprised: (i) Demographic and lifestyle variables including age, occupational rank, and smoking history; (ii) Comprehensive health metrics incorporating anthropometric measurements (height, weight, blood pressure), biochemical profiles (fasting glucose, hepatic and renal function panels, complete blood count), and specialized assessments (ophthalmic evaluation, pure-tone audiometry, ECG, with abdominal ultrasonography mandated for seafarers ≥40 years). All procedures strictly adhered to standardized maritime occupational health protocols.

According to the Guidelines for the Prevention and Control of Overweight and Obesity in Chinese Adults and considering the characteristics of body fat distribution in Asian populations, the classification criteria for BMI are defined as follows: BMI < 18.5 kg/m^2^ is classified as underweight, 18.5–23.9 kg/m^2^ as normal weight, 24.0–27.9 kg/m^2^ as overweight, and ≥28.0 kg/m^2^ as obesity. The assessment of abdominal obesity (also known as central obesity) includes waist circumference (WC) and waist-hip ratio (WHR), with the diagnostic thresholds for WC being ≥85 cm in adult males and ≥80 cm in adult females in China; the WHR criteria are ≥0.85 in males and ≥0.80 in females, which are optimized thresholds applicable to Asian populations. Definitions employed in this study were as follows: Smoking was defined as current smoking, including daily smokers or occasional smokers, where daily smokers referred to those who smoked ≥1 cigarette per day and occasional smokers were individuals who smoked ≥1 cigarette per week on average but not on a daily basis, while non-smokers included never-smokers and former smokers. Seafarers’ positions were mainly divided into three categories: engineering, deck, and service, with the engineering category responsible for maintaining the ship’s power system, the deck category overseeing navigation safety, cargo loading/unloading, and hull maintenance, and the service category undertaking logistics support such as catering, cleaning, and financial management. This study implemented standardized blood pressure measurement: subjects rested quietly for ≥5 min in a temperature-controlled and soundproof room, after which cuff size (standard/large/extra-large) was selected based on arm circumference, and three consecutive measurements were taken on the seated non-dominant arm (with 1-min intervals) with the mean value recorded. Standard 12-lead ECG was used, with ECG abnormalities classified using a four-level priority system: (1) myocardial ischemia/infarction (including ST-T changes and pathological Q waves); (2) conduction disorders (encompassing atrioventricular/bundle branch blocks and their secondary changes); (3) arrhythmias (including sinus abnormalities and various premature beats); (4) cardiac chamber hypertrophy/others (e.g., ventricular hypertrophy). Abdominal ultrasonography was conducted by radiologists with over 5 years of experience, who were completely blinded to demographic and clinical data.

### 2.3 Statistical analysis

Data were analyzed using SPSS 26.0 software. Continuous variables were expressed as mean ± standard deviation (x̄ ±s) and compared using independent samples t-tests. Categorical variables were presented as percentages (%) and analyzed using chi-square tests. Univariate and multivariate logistic regression analyses were performed to identify independent risk factors for electrocardiographic abnormalities, with statistical significance set at *p* < 0.05 (two-tailed).

## 3 Results

### 3.1 Analysis of general and abnormal results of seafarers’ health examinations

A total of 10,174 subjects were included in this study, all of them were male, the age distribution was mainly 21–30 years old and 31–40 years old, and the distribution of job types was mainly on deck and in the engine post, as shown in Table 1. The common abnormal results of seafarers’ health checkups were: BMI abnormality, abdominal obesity, refractive error, liver function abnormality, ECG abnormality, high blood pressure and high fasting blood glucose. Abnormal ECG findings in seafarers are mainly characterized by myocardial ischemia/infarction (accounting for 31.47%) and arrhythmias (accounting for 46.20%), with details presented in Table 2.

**Table 1:** General and unusual conditions of seafarers’ health examinations.

Characteristic		Number of persons (*n*)	Composition ratio (%)
Total		10,174	100.0
Age (years)	≤20	307	3.0
	21 ~ 30	1969	19.4
	31 ~ 40	4,563	44.8
	41 ~ 50	2,106	20.7
	51 ~ 60	1,229	12.1
Duties	Engine Department	4,272	42.0
	Deck Department	4,896	48.1
	Service Department	1,006	9.9
Body Mass Index (BMI)	Underweight	372	3.7
	Normal weight	4,570	44.9
	Overweight	4,342	42.7
	Obese	890	8.7
Smoking history	No	5,949	58.5
	Yes	4,225	41.5

**Table 2:** Analysis of abnormal results of seafarers’ medical examinations.

Characteristic	Number of persons (*n*)	Composition ratio (%)
Abnormal BMI	5,604	55.1
Abdominal obesity (waist circumference ≥90 cm or waist-to-hip ratio >0.9)	4,118	40.5
High fasting blood glucose (fasting blood glucose > 6.1 mmol/L)	1,175	11.5
High blood pressure (systolic ≥ 140 mmHg or diastolic ≥ 90 mmHg)	1,427	14.0
Abnormal liver function	2,343	23.0
High total bilirubin	1,340	13.2
High aspartate aminotransferase (AST)	586	5.8
High alanine aminotransferase (ALT)	1,056	10.4
ECG abnormality	2,634	25.9
Myocardial ischemia/infarction (including ST-T changes and pathological Q waves, etc.)	829	31.47
Conduction disorders (including atrioventricular/bundle branch blocks and their secondary changes, etc.)	436	16.55
Arrhythmias (including sinus abnormalities, various types of premature beats, etc.)	1,217	46.20
Arrhythmias (including sinus abnormalities, various types of premature beats, etc.)	152	5.78
Hematological abnormality	950	9.3
High white blood cell count	542	5.3
High red blood cell count	240	2.4
Low hemoglobin count	196	1.9
High platelet count	72	0.7
Abnormal urine routine	874	8.6
Positive for occult blood in urine	756	7.4
Positive urine protein	174	1.7
Refractive error	2,884	28.3
Hearing loss	641	6.3

### 3.2 Abnormal ultrasound findings in seafarers over 40 years of age

In this study, a total of 3,628 seafarers over 40 years of age underwent ultrasound examination of the liver, gallbladder, pancreas, spleen and kidneys, and a total of 2,897 (79.9%) had abnormal ultrasound results. Among them, fatty liver and urinary stones were more common, and multiple ultrasound abnormalities could exist at the same time, as shown in Table 3.

**Table 3:** Analysis of abnormal results of medical examinations of seafarers over 40 years of age.

Characteristic	Number of persons (*n*)	Composition ratio (%)
Color ultrasound (for those aged 40 and over)	2,897	79.9
Fatty liver	1,588	43.7
Kidney stones, kidney crystals	955	26.3
Liver cyst	681	18.7
Renal cyst	518	14.2
Gallbladder polyps, gallbladder polypoid lesions	409	11.2
Gallbladder stones, gallbladder intermural stones	273	7.5
Others	196	5.4

### 3.3 Univariate analysis of ECG abnormalities

The results related to seafarers’ health check-ups were analyzed according to whether the ECG results were abnormal or not, in which age, position, BMI, abdominal obesity, smoking history, blood pressure and fasting blood glucose levels were associated with abnormal ECG results, and the difference was statistically significant (*p* < 0.05) (Table 4).

**Table 4:** Univariate analysis of ECG abnormalities.

Characteristic	Quorum	Electrocardiogram (ECG) abnormality		χ^2^ value	*p-value*
		Number of examples	Proportion (%)		
Age				64.078	<0.001
≤20	307	58	18.9		
21 ~ 30	1969	492	25.0		
31 ~ 40	4,563	1,078	23.6		
41 ~ 50	2,106	594	28.2		
51 ~ 60	1,229	412	33.5		
Duties				13.466	0.001
Engine Department	4,272	1,126	27.2		
Deck Department	4,896	1,206	24.6		
Service crew	1,006	302	30.0		
BMI				8.497	0.037
Normal weight	4,570	1,157	25.3		
Underweight	372	113	30.4		
Overweight	4,342	1,109	25.5		
Obese	890	255	28.7		
Abdominal obesity				15.678	<0.001
No	6,056	1,482	24.5		
Yes	4,118	1,152	28.0		
Smoking history				647.888	<0.001
No	5,949	986	16.6		
Yes	4,225	1,648	39.0		
Fasting blood glucose (FBG)				32.738	<0.001
Normal	8,999	2,249	25.0		
Elevated	1,175	385	32.7		
Hypertension status				69.121	<0.001
Normal	8,747	2,137	24.4		
Elevated	1,427	497	34.8		
Liver function				1.608	0.205
Normal	7,831	2051	26.2		
Abnormal	2,343	583	24.9		
Complete blood count (CBC)				1.559	0.212
Normal	9,224	2,372	25.7		
Abnormal	950	262	27.6		
Urinalysis				0.257	0.612
Normal	9,300	2,414	26.0		
Abnormal	874	220	25.2		
Hearing level				3.488	0.062
Normal	9,533	2,448	25.7		
Hearing impairment	641	186	29.0		
Visual acuity				0.000	0.986
Normal	7,290	1887	25.9		
Refractive error	2,884	747	25.9		

### 3.4 Multivariable analysis of ECG abnormalities

A multivariable logistic regression analysis was performed with the dependent variable of whether the ECG results were abnormal, and the independent variables of age, job title, BMI and other factors that were statistically significant (*p* < 0.05) in the univariate analysis were included as independent variables. Multivariate logistic regression analysis revealed that elevated fasting blood glucose (OR = 1.154, 95% CI: 1.001–1.330, *p* < 0.05), elevated blood pressure (OR = 1.196, 95% CI: 1.048–1.365, *p* < 0.05), and smoking (OR = 3.290, 95% CI: 2.988–3.623, *p* < 0.05) were all independent risk factors for ECG abnormalities in seafarers’ physical examinations, with details presented in Table 5.

**Table 5:** Multivariable logistic regression analysis of ECG abnormalities.

Characteristic	B-value	SE value	Wald χ^2^ value	*p*-value	OR (95% CI)
Age					
≤20					1.000
21 ~ 30	0.155	0.159	0.954	0.329	1.168 (0.855 ~ 1.596)
31 ~ 40	−0.054	0.155	0.122	0.727	0.947 (0.699 ~ 1.284)
41 ~ 50	0.045	0.160	0.080	0.778	1.046 (0.765 ~ 1.432)
51 ~ 60	0.547	0.165	11.033	0.001	1.728 (1.251 ~ 2.386)
Duties					
Engine Department					1.000
Deck Department	−0.035	0.051	0.472	0.492	0.965 (0.873 ~ 1.067)
Service crew	0.151	0.081	3.443	0.064	1.163 (0.992 ~ 1.364)
BMI					
Underweight					1.000
Normal weight	0.342	0.124	7.588	0.006	1.408 (1.104 ~ 1.795)
Overweight	−0.145	0.058	6.230	0.013	0.865 (0.772 ~ 0.969)
Obese	−0.050	0.097	0.273	0.601	0.951 (0.787 ~ 1.149)
Abdominal obesity					
No					1.000
Yes	0.080	0.059	6.230	0.174	1.084 (0.965 ~ 1.217)
Fasting blood glucose (FBG)					
Normal					1.000
Elevated	0.143	0.072	3.902	0.048	1.154 (1.001 ~ 1.330)
Hypertension status					
Normal					1.000
Elevated	0.179	0.067	7.091	0.008	1.196 (1.048 ~ 1.365)
Smoking history					
No					1.000
Yes	1.191	0.049	586.963	<0.001	3.290 (2.988–3.623)
Constant	−1.739	0.155	125.792	-	-

## 4 Discussion

### 4.1 Analysis of the current high prevalence of diseases among seafarers and their causes

The seafarers in the results of this study in the order of disease detection rate were abnormal BMI, abdominal obesity, refractive error, abnormal liver function, abnormal ECG, high blood pressure, high fasting glucose, abnormal blood count, abnormal urine count, and hearing loss. In 2018, the prevalence of overweight and obesity among Chinese residents over 60 years old was 36.6 and 13.6%, respectively, both of which showed a continuous upward trend (1), while the detection rate of overweight and obesity among seafarers in this study was 42.7 and 8.7%, and the prevalence rate of abdominal obesity was 40.5%. BMI abnormality is closely related to a variety of chronic diseases such as diabetes mellitus, hypertension and so on (2–4), and at the same time Overweight and obesity also have a certain correlation with work ability (2). Strengthening weight management can help to better improve seafarers’ body coordination and sensitivity, and reduce the risk of accidents at sea. The results of the 2012–2015 national hypertension survey (4) showed that the prevalence of hypertension among adults in China was 23.2%, and in the current study the percentage of high blood pressure was 14.0% (2). A related study (5) states that the prevalence of diabetes mellitus globally is 9.3% in 2019 and the prevalence of pre-diabetes mellitus is increasing year by year, and the percentage of elevated fasting glucose in this study was 11.5% (5). In this study, the prevalence of refractive error and hearing loss among seafarers was 28.3 and 6.3%, respectively. The prevalence of hearing loss in the seafarer population is higher than in the general population (6), with a higher proportion of seafarers in engine positions experiencing hearing loss, and hearing loss is mainly dominated by high-frequency hearing impairment, with the level of hearing loss related to the duration of noise exposure, length of service, and so on (6).

In this study, ultrasound examination was mainly for men over 40 years old, and the rate of abnormal liver, gallbladder, pancreas, spleen and double kidney ultrasound results reached 79.9%, mainly fatty liver and kidney stones, in which a variety of abnormal ultrasound results can co-exist, while urinary stones are mostly concurrently with abnormal urinary routine, and fatty liver is often combined with abnormal liver function and gallbladder stones. With changes in lifestyle and dietary outcomes, the prevalence of fatty liver is currently on the rise. Some studies have pointed out that the prevalence of fatty liver increases with age, and its occurrence is closely related to gender, alcohol consumption, BMI level, blood glucose, blood pressure, and triglyceride level, etc. (7–9), and appropriate exercise can help reduce body weight and improve liver function, which can effectively prevent the occurrence of fatty liver (8). Urinary stones have been a prevalent disease among seafarers and one of the main causes of emergency rescue incidents at sea. In the present study, the prevalence of kidney stones and gallbladder stones was relatively high at 26.3 and 7.5%, respectively. There are also a large number of studies (10–12) pointing out that abnormal BMI, dietary habits, average daily water intake, etc. are closely related to urinary stones.

### 4.2 Analysis of ECG abnormalities and their influencing factors among seafarers

Given that seafarers are chronically exposed to the unique maritime working environment characterized by factors such as high-intensity work stress, irregular work-rest schedules, and confined living spaces, their cardiovascular health risks are significantly elevated compared to the general population. As a fundamental tool for evaluating cardiac electrical activity, the ECG serves as an indispensable screening modality in seafarer health assessments, offering not only early detection of asymptomatic cardiovascular disease but also enabling timely intervention for critical conditions such as myocardial infarction and arrhythmias. This study revealed an overall ECG abnormality detection rate of 25.9%, with myocardial ischemia/infarction (accounting for 31.47% of abnormalities) and arrhythmias (accounting for 46.20% of abnormalities) being the predominant types. This data suggests a notably high prevalence of potential disturbances in cardiac electrical activity within this occupational group; such abnormalities often serve as early indicators of underlying cardiovascular disease (e.g., myocardial ischemia may signal coronary insufficiency, while arrhythmias may be associated with cardiac autonomic dysfunction or organic pathology), and their timely detection is crucial for preventing severe cardiovascular events such as acute myocardial infarction and sudden cardiac death. The study identified elevated fasting blood glucose, elevated blood pressure, and smoking as independent risk factors for ECG abnormalities among seafarers, aligning with findings from numerous previous studies conducted in both general and occupational populations (13–21), suggesting that these factors constitute core mechanisms driving cardiac electrical disturbances in seafarers as well. Concurrently, although factors such as age, obesity, and abdominal obesity were not identified as independent risk factors in this specific analysis, their established status as recognized cardiovascular disease indicators is well-documented, with extensive research (2, 3, 17, 22, 23) confirming associations between these factors and alterations in cardiac structure and function, such as left ventricular hypertrophy and abnormal myocardial metabolism.

The impact of elevated fasting blood glucose on cardiac electrical activity involves multifaceted mechanisms: On one hand, hyperglycemia may induce microvascular dysfunction, impairing myocardial microcirculatory perfusion and resulting in myocardial ischemia and hypoxia; on the other hand, electrolyte disturbances triggered by hyperglycemia can directly disrupt myocardial depolarization and repolarization processes, ultimately manifesting as electrocardiographic abnormalities (24). The association between hypertension and electrocardiographic abnormalities is predominantly mediated by adaptive changes in cardiac structure and function. Chronically elevated blood pressure significantly increases cardiac afterload, leading to compensatory left ventricular hypertrophy and accelerated progression of coronary atherosclerosis, which collectively induce myocardial ischemia (25, 26). Concurrently, hypertension heightens arrhythmia risk by compromising myocardial electrophysiological stability and directly contributes to severe cardiovascular events such as stroke and heart failure (17–20). The cardiovascular detriment of smoking manifests through dual disruption of myocardial oxygen supply–demand balance (21): Nicotine stimulates catecholamine release via sympathetic activation, increasing heart rate and myocardial oxygen consumption, which may induce sinus tachycardia or premature beats. Concurrently, carbon monoxide generated from smoking competitively binds to hemoglobin, impairing oxygen-carrying capacity and exacerbating myocardial ischemia or injury, ultimately presenting as electrocardiographic abnormalities including ST-T segment alterations (21, 23). Crucially, these three risk factors exhibit synergistic interactions—hyperglycemia potentiates vascular damage in hypertensive patients, while smoking amplifies the deleterious effects of both hyperglycemia and hypertension (21, 23–25). Such interactions multiplicatively escalate cardiovascular risk, underscoring the imperative for multifactorial intervention strategies.

### 4.3 Potential risk factors not included in the study and their cardiovascular implications

Although this study delineated the independent contributions of dysglycemia, hypertension, and smoking, the complexity of cardiovascular risk among seafarers extends beyond these established determinants. Unassessed variables—including alcohol consumption, physical exercise, work schedules, fatigue, psychological stress, and sleep patterns—may directly or indirectly modulate electrocardiographic findings through multifactorial pathways and warrant thorough investigation. The cardiac effects of alcohol exhibit dose-dependent duality, with seafarers potentially demonstrating elevated consumption rates due to occupation-specific factors (e.g., maritime socialization, stress mitigation) (26). While low-to-moderate intake may transiently lower blood pressure via vasodilation, chronic excessive consumption induces direct cardiomyocyte injury, leading to myocardial fibrosis and electrophysiological disturbances that manifest as arrhythmias on ECG (26, 27). Concurrently, alcohol and tobacco synergistically stimulate sympathetic activation, significantly augmenting myocardial oxygen demand and exacerbating electrical abnormalities. Furthermore, confined maritime environments coupled with limited exercise facilities despite high occupational exertion contribute to prevalent physical inactivity among seafarers (21, 23, 26). Physical inactivity impairs insulin sensitivity, thereby exacerbating dysglycemia, while concurrently reducing vascular compliance and elevating blood pressure—all of which indirectly disrupt cardiac electrophysiology through integrated hemodynamic and metabolic pathways.

Prolonged working hours and chronic fatigue represent significant occupational health threats for seafarers. Empirical studies indicate a statistically significant positive correlation between extended onboard duty duration and fatigue levels (27–29). Persistent fatigue potentiates elevated catecholamine secretion, which may trigger tachycardia, blood pressure lability, and ultimately electrocardiographic abnormalities including arrhythmias (30). Furthermore, fatigue impairs glycemic control (e.g., through insulin resistance), establishing a bidirectional exacerbation cycle with hyperglycemia (24, 29). Seafarers exhibit significantly higher rates of psychological distress and sleep disorders compared to the general population (30). Chronic stress elevates cortisol levels, inducing blood pressure fluctuations and increased myocardial oxygen demand, while sleep disorders—particularly obstructive sleep apnea (OSA)—provoke nocturnal hypoxemia and heighten arrhythmia susceptibility via sympathetic activation (26, 31). Crucially, sleep quality demonstrates significant correlations with both somatic symptoms and mental health in this cohort, with seafarers experiencing somatization showing elevated vulnerability to sleep disturbances (31). Persistent exposure to vessel noise (e.g., engine/mechanical operations) constitutes an entrenched environmental hazard; chronic noise exposure elevates blood pressure through sympathetic stimulation and directly compromises myocardial electrophysiological stability, leading to electrocardiographic abnormalities (6, 32, 33). Additionally, shipboard vibration may accelerate atherosclerosis by impairing endothelial function, synergistically potentiating hypertension and myocardial ischemia (32).

### 4.4 Recommendations for the health management of common seafarer illnesses

The COVID-19 pandemic has inflicted unprecedented disruption on global shipping industries. Seafarers—the essential workforce enabling 90% of worldwide merchandize transport—have suffered profound deterioration in occupational viability, physical/mental health, and rights protection. The pandemic’s impact on this cohort manifests as interconnected multidimensional challenges: (i) Global travel restrictions incapacitated crew-change systems, precipitating pervasive contract extensions. Compounded by vessel understaffing, these conditions triggered surging workloads and cumulative fatigue; (ii) Confined onboard environments amplified viral transmission risks, while port access restrictions disrupted medication supply and routine health screenings—severely compromising management of hypertension, dysglycemia, and other chronic conditions. Furthermore, the synergistic effects of psychological distress (anxiety/isolation), physical inactivity, and dietary deterioration substantially elevated cardiovascular risks, establishing potential associations with early pathological indicators such as electrocardiographic abnormalities; (iii) Most nations lacked tailored entry exemptions and social security mechanisms, resulting in crisis-level crew-change impasses and inadequate medical coverage. This not only intensified occupational insecurity among seafarers but also undermined long-term workforce stability in the shipping industry. These systemic deficiencies underscore the global maritime sector’s institutional gaps in safeguarding seafarer rights and health during public health emergencies.

Collectively, an end-to-end institutional framework must be established across legislative, healthcare, managerial, and preventive dimensions to holistically safeguard seafarers’ rights. Specifically for managing occupationally prevalent diseases, strategic implementation should focus on: strengthening legal safeguards through codifying core entitlements—including rest periods, medical care, and repatriation—in maritime labor legislation; optimizing integrated healthcare systems by incorporating telemedicine, health literacy programs, and emergency guidance, while mandating vessels transporting hazardous cargo to stock specific antidotes and protective equipment alongside regular occupational disease screenings; implementing tiered chronic disease management that categorizes seafarers by condition severity and risk profiles, providing prioritized medical access, medication optimization, and lifestyle interventions to prevent emergencies, delay disease progression, and enhance quality of life; and enhancing mental health support with safety training to elevate health awareness through routine check-ups and disease education, while mitigating risks via tailored interventions such as custom-fitted hearing protection and diversified exercise equipment—all aimed at reducing maritime morbidity and improving sustainable health outcomes.

This study investigated seafarer health examination profiles, delineated prevalent diseases within this occupational cohort, and identified significant risk factors for electrocardiographic abnormalities—findings with substantial implications for cardiovascular disease prevention among seafarers. Concurrently, it provides partial scientific foundations for public health authorities to formulate targeted health policies and interventions, thereby contributing to sustainable maritime industry development. However, several methodological limitations warrant objective critique: First, incomplete inclusion of key variables (e.g., work schedules, physical activity, psychological stress, alcohol consumption) may introduce selection bias, potentially overestimating/underestimating effects of identified factors like fasting glucose and hypertension, or even causing confounding. Second, the cross-sectional design without longitudinal assessment precludes tracking dynamic progression of ECG abnormalities (e.g., evolution into clinical cardiovascular events), limiting causal inference. Third, insufficient statistical rigor—particularly absent multicollinearity diagnostics—could compromise parameter estimation stability. Collectively, these constraints necessitate future studies with expanded variable coverage, prospective designs to monitor disease trajectories, and enhanced analytical methods to robustly elucidate risk determinants and causal pathways of seafarer ECG abnormalities.

## 5 Conclusion

This study identified prevalent seafarer health abnormalities: BMI deviations, abdominal adiposity, refractive errors, hepatic dysfunction, ECG abnormalities, elevated BP, impaired fasting glucose, hematologic/urinary anomalies, and hearing impairment. Specifically, ECG abnormalities manifested as myocardial ischemia/infarction (31.47%) and arrhythmias (46.20%). Elevated fasting glucose, hypertension, and smoking were significant ECG risk factors. Enhancing health awareness and implementing structured follow-up services may reduce chronic disease incidence, improve physical fitness, and support sustainable maritime operations.

## Data Availability

The raw data supporting the conclusions of this article will be made available by the authors, without undue reservation.
